# Identification of cCMP- and cUMP-binding proteins using cCMP and cUMP coupled to agarose and biotin matrices

**DOI:** 10.1371/journal.pone.0333904

**Published:** 2025-10-14

**Authors:** Sabine Wolter, Martin Neumann, Theresa Koenig, Tina Hagedorn, Frank Schwede, Andreas Pich, Roland Seifert

**Affiliations:** 1 Institute of Pharmacology, Hannover Medical School, Hannover, Germany; 2 Biolog Life Science Institute GmbH & Co. KG, Bremen, Germany; 3 Institute of Toxicology and Research Core Unit Proteomics, Hannover Medical School, Hannover, Germany; University of Minnesota, The Hormel Institute, UNITED STATES OF AMERICA

## Abstract

There is increasing evidence for a role of cyclic CMP (cCMP) and cUMP as second messengers. In a recent study we showed that cCMP activates both purified cGMP-dependent protein kinase Iɑ (PKGIɑ) and cAMP-dependent protein kinase (PKA) isoenzymes with the regulatory subunits RIɑ and RIIɑ. PKARIɑ was identified as a cCMP-binding protein using cCMP coupled to agarose by immunoblotting and PKARIIɑ by MS analytic. In this study, we discovered PKARIɑ, PKARIIɑ and PKG as cCMP- and also cUMP-binding partners using cCMP- and cUMP-agarose. For the first time as well as cCMP and cUMP coupled to biotin matrices was used and from mouse lung tissue, A549 and HeLa cell lysates the identical proteins were also identified as cCMP and cUMP binding proteins. In proteomic approaches, three isoforms of PKG (PKGI, PKGIβ and PKGII) were identified as cCMP- and cUMP-binding proteins from mouse lung tissue. Here we show the binding of cCMP and cUMP to the most prominent target proteins PKA and PKG of the second messengers cAMP and cGMP. These results point to an impact for cCMP and cUMP as non-canonical second messengers in signal transduction pathways like cAMP and cGMP. Furthermore, the results show that the agarose matrices and also the cNMP botin matrices are excellent tools for identifying new binding partners for cCMP and cUMP.

## Introduction

The cyclic purine nucleotides cAMP and cGMP are established second messengers, which are referred to as canonical second messengers. In order to qualify as second messenger, a molecule must fulfill the following four criteria: (1) Generation by a first messenger regulated enzyme; (2) activation of specific effector proteins; (3) defined biological functions; (4) specific inactivation mechanisms [1,2]. Several second messengers fulfill two additional criteria: (a) Mimicry by membrane-permeable second messenger analogs; and (b) mimicry by bacterial toxins [1,2]. cAMP and cGMP fulfill all of the six second messenger criteria introduced above [1,2]. The cyclic pyrimidine nucleotides cCMP and cUMP are emerging second messengers, termed as non-canonical second and fulfill most of the criteria of second messengers [1,3]. cCMP and cUMP have been clearly identified in many mammalian cell lines and primary cells using mass spectrometry methods [4]. Moreover, the *Pseudomonas aeruginosa* nucleotidyl cyclase toxin ExoY effectively increases cellular cCMP and cUMP levels in an acute mouse lung infection model [5]. Among the many mammalian PDEs studied so far, cCMP is hydrolyzed by PDE7A1 with low affinity but high capacity [6], whereas until now, two PDEs have been identified which hydrolyzed cUMP, namely PDE3B and PDE9A [7,8]. However, the physiological role of cCMP and cUMP is not yet fully understood, but it was shown that cCMP and cUMP induced apoptosis in mouse lymphoma cell lines and in human erythroleukemia (HEL) cells [9]. Moreover, regulation of HCN channels by cCMP and cUMP was described [10,11]. Recently, specific cytidylyl and uridylyl cyclases have been identified in numerous bacterial species [12]. cCMP and cUMP are part of the bacterial immune system. After infection with bacteriophages specific targets are activated which lead to bacterial NAD^+^ depletion [12].

PKA in the inactive form is a tetramer of two regulatory and two catalytic subunits, whereby the regulatory domains have four isoforms, i.e. RIɑ, RIβ, RIIɑ, and RIIβ [13]. PKG is a serine/threonine protein kinase, which is potently and effectively activated by cGMP [14]. Mammalian PKGs are encoded by two genes, PKGI and PKGII [14], whereas PKGI has two isoforms, PKGIɑ and PKGIβ, which differ from each other only in the N-terminal amino acid sequence. It was shown that cCMP and cUMP can also activate the main cAMP and cGMP target molecules PKA and PKG [15,16]. Differences between the distinct subunits were detected. PKARIɑ and PKARIIɑ are activated by cCMP and cUMP, but the potency of cCMP and cUMP is lower than that of cAMP. Moreover, cCMP and cUMP are less potent and less efficacious activators of purified PKGIɑ than cGMP [15,16].

In recent studies, we used agarose matrices coupled to cCMP and to cUMP to identify other binding proteins by MS analysis. Because of the elevated cUMP and cCMP levels detected after treatment with ExoY in an acute lung mouse infection model [5] we used mouse lung tissue, A549 cells, a human alveolar basal epithelial cell line, and HeLa cells. These cells were already used by Hammerschmidt et al. [17]. Furthermore, the endogenous content of cUMP in A549 cells is relative high level, whereas HeLa cells were chosen as an example for cells with a very low content of cUMP [18]. In the present work, we conducted studies to identify further binding partners for cCMP and cUMP using biotin matrices coupled to cCMP and cUMP, respectively.

## Materials and methods

All standard chemicals were purchased from commercial sources and were all of analytical grade, unless stated otherwise.

### cCMP and cUMP coupled to agarose

**Fig 1:**
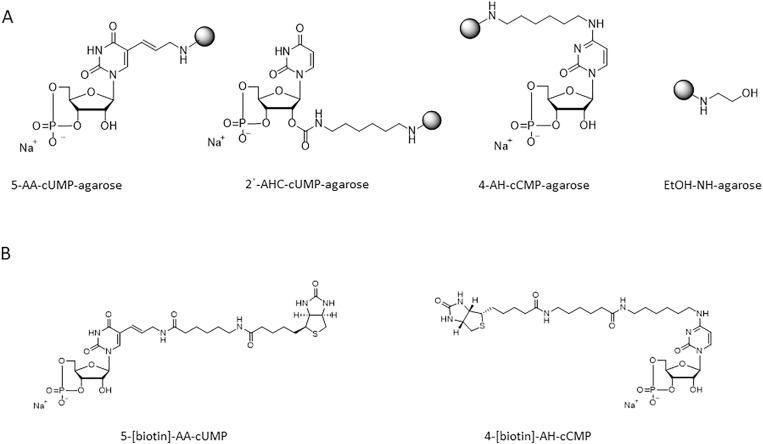
Structures of cCMP- and cUMP-agaroses, EtOH-NH-agarose and cCMP- and cUMP-biotin matrices.

N^4^-(6-aminohexyl)-cCMP-agarose (4-AH-cCMP-agarose (AH)), 2'-O-(6-aminohexylcarbamoyl)-cUMP-agarose (2ˈ-AHC-cUMP-agarose (AHC)), and 5-aminoallyl-cUMP-agarose (5-AA-cUMP-agarose (AA)) were used for affinity chromatography. As control, ethanolamine immobilized on agarose gel (EtOH-NH-agarose) was used. Detailed information is provided by Schwede et al. [19]. cNMP-agarose matrices were from Biolog Life Science Institute GmbH & Co. KG (Biolog), Bremen, Germany. Fig 1A shows the structures of the binding matrices used.

### cCMP and cUMP coupled to biotin

4-[biotin]-AH-cCMP and 5-[biotin]-AA-cUMP were used for affinity chromatography. Information about synthesis is provided in Schwede et al. [19]. cNMPs coupled to biotin matrices were from Biolog. Fig 1B shows the structures of the binding matrices used.

### Cell culture

A549 (adenocarcinomic human alveolar basal epithelial) cells were obtained from Dr. A. Munder, Clinic for Paediatric Pneumology, Allergology and Neonatology, Hannover Medical School, Hannover, Germany. HeLa cervix carcinoma cells were obtained from the American Type Culture Collection. A549 and HeLa cells were cultured with Dulbecco’s modified Eagle’s medium (high glucose) supplemented with 10% (v/v) fetal bovine serum (PAN Biotech, Aidenberg, Germany), 200 µg/ml L-glutamine, 100 U/ml penicillin, and 0.1 mg/ml streptomycin in a humidified atmosphere containing 5% (v/v) CO_2_ at 37°C.

### Cell lysates

A549 and HeLa cells were washed with ice-cold PBS and collected by centrifugation. The pellets were lysed by incubation on ice with ice-cold triton puffer (10 mM TRIS pH 7.05, 30 mM NaPPi, 1% (v/v) Triton, 50 mM NaCl, 50 mM NaF, 20 mM b-glycerophosphate, phosphatase and proteinase inhibitor cocktail (Pierce Protease and Phosphatase Inhibitor Mini Tablets; Thermo Fisher Scientific, Schwerte, Germany) for 20 min. After centrifugation for 30 min at 13000 x g at 4°C the supernatant fluid was removed and stored by −20°C for further use.

### Mouse lung tissue

BALB/c mice were obtained from Elevage Janvier (Le Genest‐Saint‐Isle, France). Animals were handled according to the “Declaration of Helsinki” and the guidelines of the local ethics committee. Mice were fed with food and tap water ad libitum, maintained at constant temperature (22°C) and housed under a light cycle of 12 h light/12 h darkness. Eight- to ten- week-old Balb/c mice were sacrified by CO_2_ suffocation followed by cervical dislocation. After bleeding by heart puncture the lungs were excised and, washed with ice-cold PBS, immediately shock-frozen in liquid nitrogen, and stored at −80°C until processing.

For protein isolation, 100–200 mg fragments of lung tissue were homogenized with 1 ml of tween lysis buffer (50 mM K_3_PO_4_, 150 mM NaCl, 0.2% (v/v) Tween20, phosphatase and proteinase inhibitor cocktail) in an Elvehjem glass tube potter. After centrifugation for 10 min at 9,000 x g at 4°C the supernatant fluid was removed and stored by −20°C for further use.

### Protein determination

The protein concentration was analyzed by the BCA (bicinchoninic acid) protein assay according to the manufacturer’s instructions (Thermo Fisher Scientific).

### Affinity chromatography

a) Agarose matrices: 4-AH-cCMP-agarose, 5-AA-cUMP-agarose, 2ˈ-AHC-cUMP-agarose and EtOH-NH-agarose (30 µl each) were equilibrated three times with triton buffer. 2 mg of A549 or HeLa cell lysate were incubated under rotation (700 x g) with agarose and control beads in a total volume of 500 µl in triton buffer completed with 100 µM IBMX (isobutyl-methylxanthine) overnight at 4°C. cCMP or cUMP at a concentration of 2 mM were added to certain samples to detect non-specific binding. Samples were centrifuged at 700 x g for 1 min at 4°C, and beads were washed five times with 500 µl triton buffer. After that, the supernatant fluid was removed completely. The procedure for the mouse lung lysates was analogous, but instead of the triton buffer tween lysis buffer was used.
b) Biotin-matrices: Strep-tactin beads (100 µl per assay; IBA, Göttingen, Germany) were equilibrated three times with triton buffer and further incubated with 4-[biotin]-AH-cCMP, 5-[biotin]-AA-cUMP, and biotin as control (50 nmol each) for 30 min at 500 rpm in a thermal mixer at room temperature. Non-coupled biotin was removed by washing three times with triton buffer. 2 mg of cell lysate or mouse tissue were incubated with the biotin-Strep-tactin-compounds in lysis buffer completed with IBMX (100 µM) and protease inhibitor cocktail (Halt; Thermo Fisher Scientific) in a total volume of 500 µl at 4°C overnight (14 rpm overhead). cCMP or cUMP at a concentration of 2 mM were added to certain samples to detect non-specific binding. Samples were centrifuged at 700 x g for 1 min at 4°C, and beads were washed five times with 500 µl buffer. After that, the supernatant fluid was completely removed. Triton lysis buffer was used for cell lysates and Tween20 lysis buffer for mouse lung tissue.

### Sample preparation for MS analysis

Proteins were eluted from the cAMP-, cUMP- or control EtOH-NH-agarose with 40 µl Roti-Load (Roth, Karlsruhe, Germany) by heating for 10 min. For alkylation of cysteine residues, 1.5 µl of an acrylamide solution 40%, (m/v) was added and incubated for 30 min at room temperature. Proteins were separated by SDS-PAGE using a gradient gel (4–20% Precise Tris-Glycine-Gel, Thermo Fisher Scientific). Proteins were stained with coomassie. After destaining and documentation by photography, protein lanes were cut into 5 pieces, shredded into cubes, and completely destained with ACN 50% (v/v) in 20 mM NH_4_HCO_3_ for 30 min at 37°C at 300 rpm followed by dehydration with 100% ACN and drying by vacuum centrifugation. Gel pieces were incubated on ice for 1 h with ACN 10% (v/v) in 20 mM NH_4_HCO_3_ containing 12 ng/µl trypsin for dehydration and subsequently at 37°C at 300 rpm over night for digestion which was stopped by adding ACN 10% (v/v) containing 0.5% (v/v) TFA. Peptide extraction was done two times by adding ACN 10% (v/v) containing 0.2% (v/v) TFA to the gel pieces. The supernatant fluids of the extracted peptides were pooled and dried by vacuum centrifugation.

Trypsin digestion of mouse lung tissue was stopped by adding 150 µl H_2_O and incubation for 30 min at room temperature. Extraction was performed two times with ACN 50% (v/v) with 5% (v/v) TFA by incubation at room temperature for 30 min. Subsequently, 100% ACN was added to the gel pieces and all supernatant fluids were pooled and dried by vacuum centrifugation. Two independent biological trials with two technical replicates were performed for mass spectrometry experiments.

### LC-MS analysis

The LC-MS analysis was performed according to the detailed description in [20]. Briefly, extracted peptides were separated by a nanoflow reversed phase chromatography system (RSLC, Thermo Fisher Scientific) consisting of a C18 PepMap column (Thermo Fisher Scientific) with a flow rate of 250 nL/min by 45°C. As eluent A 0.1% (v/v) formic acid and as eluent B 80% (v/v) ACN, 0.1% (v/v) formic acid was used. Peptides were eluted with a multistep linear gradient, starting with 96% eluent A (0.1% formic acid and 4% eluent B (0.1% formic acid, 99.9% ACN), reaching 60% eluent A and 40% eluent B in 120 min. Then, the eluent B was further increased to 75% within 10 min. After 20 min, eluent B was further increased to 90% in 10 min. This concentration was maintained for 10 min, before eluent A was increased and eluent B was reduced to the starting conditions. Finally, the column was regenerated at starting conditions for 15 min.

The outlet of the column was directly connected to the nanoelectrospray source of an LTQ Orbitrap Velos (Thermo Fisher Scientific).

### LTQ orbitrap velos

Eluted peptides were ionized with an emitter voltage of 1.2 kV. MS-overview scans were created in the profile mode in the range from 300–1600 m/z with a resolution of 60.000 at 400 m/z. The minimal signal threshold was set to 2,000 counts, 10 of the most intensive peptides with a charge of two or three were submitted to collision-induced dissociation (CID) fragmentation with an activation time of 10 ms and normalized collision energy of 38%. Dynamic exclusion duration was set to 70 s. Fragmented peptides were detected in a dual pressure linear ion trap.

### Protein identification

Raw data were searched with Proteome Discoverer software (Thermo Fisher Scientific) using the Sequest algorithm and human and mice entries of Uniprot data base. Peptides and proteins were stated identified by a false discovery rate of < 0.05 using a target decoy strategy and proteins were identified if at least two peptides were found for a single protein group.

### Western blotting

Proteins were eluted from the cCMP-, cUMP-agarose or cCMP-, cUMP-biotin coupled to strep-tactin beads with 40 µl Roti-Load (Roth) by heating for 10 min. Proteins were separated by SDS-PAGE using a gel containing 7.5% or 10% (m/v) acrylamide. Following transfer to a nitrocellulose membrane, the proteins were analyzed with first antibodies (ɑ-PKARIɑ (sc-136231, 1:500, Santa Cruz, Heidelberg, Germany), ɑ-PKARIIɑ (sc-908, 1:500, Santa Cruz), ɑ-PKG (ADI-KAP-PK005-D, 1:1000, Enzo Life Science, Lörrach, Germany) and anti-mouse IgG- or anti-rabbit IgG-linked to horseradish peroxidase (Cell Signaling, Frankfurt/M, Germany) as second antibodies. The ɑ-PKARIɑ antibody also recognizes the RIβ isoform of PKA. Blots were developed with WesternSure Premium chemiluminescent substrate (Li-Cor, Bad Homburg, Germany) with the C-Digit Blot scanner. Evaluation was done with the Image Studio Lite software (version 4.0). Two biological assays were performed for western blot analysis.

## Results

### Identification of different isoforms of PKG, PKARIɑ, and PKARIIɑ as cCMP- and cUMP binding proteins using agarose matrices by MS analysis

**Fig 2:**
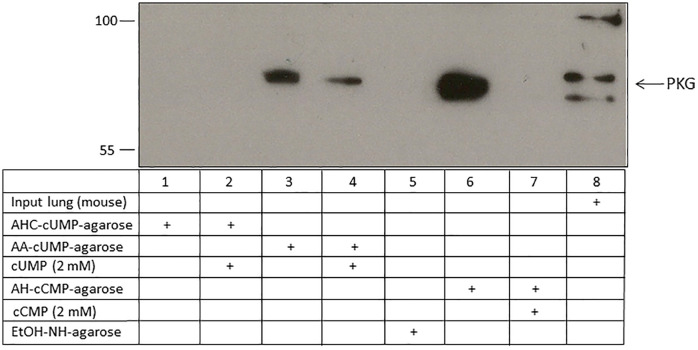
Binding of PKG from mouse lung tissue to cCMP and cUMP. Lysate of mouse lung tissue was incubated with 2ˈ-AHC-cUMP-agarose, 5-AA-cUMP-agarose, 4-AH-cCMP-agarose and EtOH-NH-agarose (control agarose). In competition assays, 2 mM cUMP was added to the cUMP-agarose samples, and 2 mM cCMP was added to the cCMP-agarose samples. Input designates mouse lung tissue lysate before incubation. PKG was detected by immunoblotting.

Using 5-AA-cUMP-agarose, three isoforms of PKG – PKGI, PKGIβ, and PKGII were identified by MS analysis as cUMP-binding proteins from mouse lung tissue with high protein coverage and several unique peptides (Table 1a; S1 Fig). The MS spectrum for PKG is shown in S1 Fig. Further, using 4-AH-cCMP-agarose all three isoforms of PKG could be determined as cCMP-binding proteins by MS analysis from mouse lung tissue, and PKGI from HeLa cells (Table 1b). Protein coverage for PKGI isoforms was much higher than the coverage for the PKGII isoform from mouse lung tissue or the PKGI isoform in HeLa cells. For mouse lung tissue, binding of PKG to cCMP and cUMP was also verified by western blotting (Fig 2; S3 Fig). No binding of PKG isoforms to the EtOH-NH-agarose was detected.

**Table 1:** a. Identification of PKG isoforms by MS analytics using 5-AA-cUMP-agarose from mouse lung tissue: Summary.

Accession number	Protein	Isoform	Gene	Coverage [%]	# Unique peptides	# Peptides	Source
P0C605	cGMP-dependent protein kinase	1	Prgk1	44.3	8	33	lung, mouse
P0C605	cGMP-dependent protein kinase	1	Prgk1	44.4	5	31	lung, mouse
E9QPH0	cGMP-dependent protein kinase	2	Prkg2	26,3	20	20	lung, mouse
E9QPH0	cGMP-dependent protein kinase	2	Prkg2	17,3	15	15	lung, mouse
P0C605-2	cGMP-dependent protein kinase	1	Prgk1	42.6	5	31	lung, mouse
P0C605-2	cGMP-dependent protein kinase	1	Prgk1	39,7	2	28	lung, mouse
**b. Identification of PKG isoforms by MS analytics using 4-AH-cCMP-agarose from mouse lung tissue and HeLa cells: Summary**							
P0C605	cGMP-dependent protein kinase	1	Prgk1	44.4	10	33	lung, mouse
E9QPH0	cGMP-dependent protein kinase	2	Prkg2	28.2	23	23	lung, mouse
P0C605-2	cGMP-dependent protein kinase	1 b	Prgk1	39.1	4	28	lung, mouse
Q13976	cGMP-dependent protein kinase	1	Prgk1	3.2	2	2	HeLa cells

n = 2. No binding from isoforms of PKG to EtOH-NH-agarose was detected.

n = 2. No binding from isoforms of PKG to EtOH-NH-agarose was detected.

**Fig 3:**
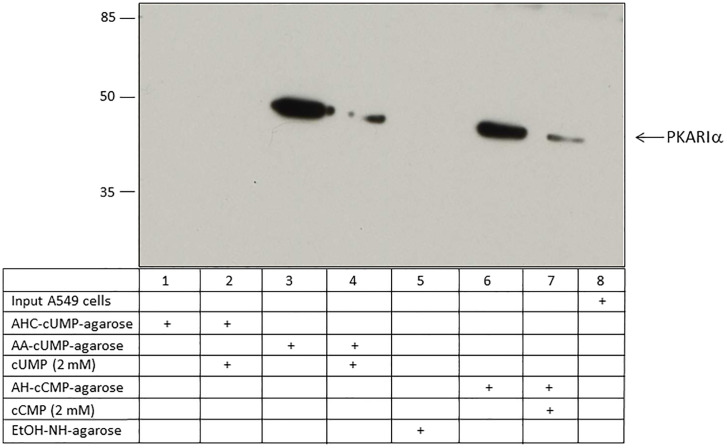
Binding of PKARIɑ from A549 cells to cCMP- and cUMP-agarose. Cell lysate of A549 cells was incubated with 2ˈ-AHC-cUMP-agarose, 5-AA-cUMP-agarose, 4-AH-cCMP-agarose and EtOH-NH-agarose (control agarose). In competition assays, 2 mM cUMP was added to the cUMP-agarose samples, and 2 mM cCMP was added to the cCMP-agarose samples Input designates A549 cell lysate before incubation. PKARIɑ was detected by immunoblotting.

PKARIɑ was identified as a cUMP-binding protein using 5-AA-cUMP-agarose and 2ˈ-AHC-cUMP-agarose in A549 cells, HeLa cells and mouse lung tissue (Table 2a; S2 Fig). The MS spectrum for PKARIɑ is shown in S2 Fig. Also, PKARIɑ was confirmed as cCMP-binding protein in A549 cells, HeLa cells and mouse lung tissue using 4-AH-cCMP-agarose (Table 2a). The coverage and number of unique peptides from experiments with 2ˈ-AHC-cUMP-agarose were much lower than the coverage and number of unique peptides from experiments with 5-AA-cUMP- or 4-AH-cCMP-agarose. Partially, these results could be confirmed by western blot analysis of A549 cells (Fig 3; S3 Fig). No binding of the PKARIɑ proteins was detected by using 2ˈ-AHC-cUMP-agarose. No expression of PKARIɑ was detectable in A549 cell lysate – input lane.

**Table 2:** a. Identification of PKARIɑ by MS analytics from mouse lung tissue, A549 and HeLa cells: Summary.

Agarose	Coverage [%]	# Unique peptides	# Peptides	Source
AA-cUMP	36.2	13	14	A549 cells
AA-cUMP	44.6	17	21	A549 cells
AH-cCMP	37.0	14	15	A549 cells
AH-cCMP	26.7	10	10	A549 cells
AHC-cUMP	16.0	5	5	A549 cells
AA-cUMP	45.1	21	26	HeLa cells
AH-cCMP	51.4	22	26	HeLa cells
AHC-cUMP	26.5	8	10	HeLa cells
AHC-cUMP	21.0	7	7	HeLa cells
AA-cUMP	48.8	18	24	lung, mouse
AA-cUMP	48.8	21	27	lung, mouse
AH-cCMP	46.7	18	23	lung, mouse
AHC-cUMP	7.4	2	2	lung, mouse
AHC-cUMP	17.9	7	7	lung, mouse
**b. Identification of PKARIIɑ by MS analytics from mouse lung tissue, A549 and HeLa cells: Summary**				
**Agarose**	**Coverage [%]**	**# Unique peptides**	**# Peptides**	**Source**
AA-cUMP	54.5	21	23	A549 cells
AA-cUMP	65.6	24	27	A549 cells
AH-cCMP	62.9	23	26	A549 cells
AA-cUMP	68.8	28	28	HeLa cells
AH-cCMP	68.6	26	26	HeLa cells
AH-cCMP	75.3	32	32	HeLa cells
AHC-cUMP	44.5	15	15	HeLa cells
AA-cUMP	71.6	29	34	lung, mouse
AA-cUMP	66.9	29	35	lung, mouse
AH-cCMP	70.4	24	28	lung, mouse
AHC-cUMP	5.0	2	2	lung, mouse
AHC-cUMP	13.9	6	6	lung, mouse

n = 2. Partially, binding of PKARIɑ to EtOH-NH-agarose was detected.

n = 2. Partially, binding of PKARIɑ to EtOH-NH-agarose was detected.

**Fig 4:**
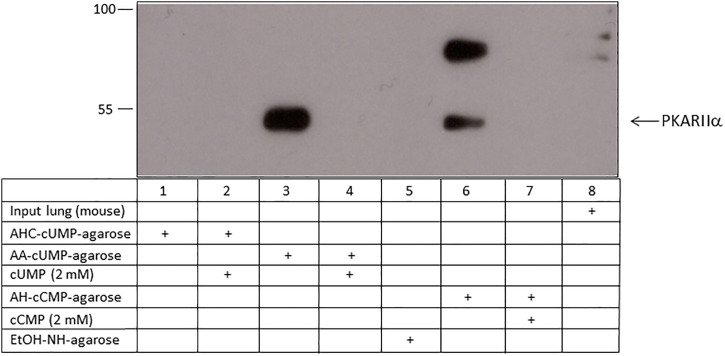
Binding of PKARIIɑ from mouse lung tissue to cCMP and cUMP. Lysate of mouse lung tissue was incubated with 2ˈ-AHC-cUMP-agarose, 5-AA-cUMP-agarose, 4-AH-cCMP-agarose and EtOH-NH-agarose (control agarose). In competition assays 2 mM cUMP was added to the cUMP-agarose samples, and 2 mM cCMP was added to the cCMP-agarose samples. Input designates mouse lung tissue lysate before incubation. PKARIIɑ was detected by immunoblotting.

Using 5-AA-cUMP-agarose, PKARIIɑ was identified from A549 cells, HeLa cells and mouse lung tissue. Using 2ˈ-AHC-cUMP-agarose PKARIɑ was also detectable in HeLa cells and mouse lung tissue as cUMP-binding protein (Table 2b). Starting from A549 cells, HeLa cells and mouse lung tissue PKARIIɑ was also confirmed by MS analytic as cCMP-binding partner using agarose coupled to cCMP (Table 2b). However, also binding from PKARIɑ and PKARIIɑ to the EtOH-NH-agarose was found. The results for mouse lung tissue were verified by western blotting (Fig 4). No binding was detected when using 2ˈ-AHC-cUMP-agarose. No expression of PKARIIɑ was detectable in mouse lung tissue lysate – input lane.

### Identification of PKARIɑ, PKARIIɑ, and PKG as cCMP- and cUMP binding proteins by immunoblotting

In A549 cells, PKARIɑ was identified as a cUMP- and cCMP-binding protein using 5-AA-cUMP-agarose and 4-AH-cCMP-agarose (Fig 3; S4 Fig). Competition experiments showed a lower binding than approaches without cCMP or cUMP. 2ˈ-AHC-cUMP-agarose was not able to precipitate PKARIɑ. No binding of PKARIɑ to the control agarose (EtOH-NH-agarose) was detected. Based on lung tissue as starting material, comparable results were obtained for binding of cUMP and cCMP to PKG and PKARIIɑ (Figs 2, 4; S3, S5 and S5A Figs). Further, 2ˈ-AHC-cUMP-agarose failed to precipitate PKARIIɑ or PKG from mouse lung tissue.

### Identification of PKARIɑ as cCMP- and cUMP-binding partners using biotin coupled to cCMP and cUMP

**Fig 5:**
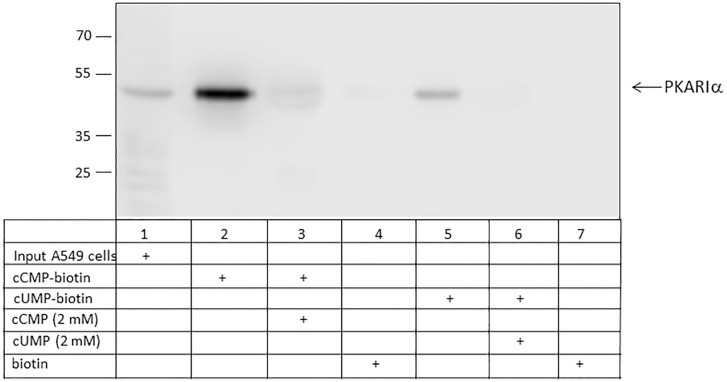
Binding of PKARIɑ from A549 cells to cCMP and cUMP using biotin-matrices. cUMP-biotin, cCMP-biotin and biotin as control were incubated with strep-tactin beads and and afterwards incubated with cell lysate of A549 cells. In competition assays 2 mM cUMP and 2 mM cCMP was added, respectively. Input designates A549 cell lysate before incubation. PKARIɑ was detected by immunoblotting.

**Fig 6:**
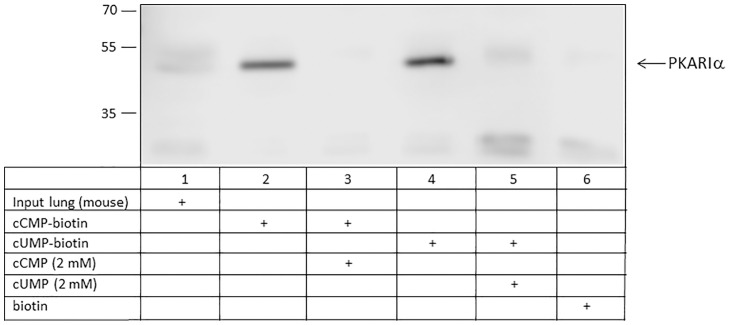
Binding of PKARIɑ from mouse lung tissue to cCMP and cUMP using biotin matrices. cUMP-biotin, cCMP-biotin and biotin as control were incubated with strep-tactin beads first and with lysate of mouse lung tissue afterwards. In competition assays, 2 mM cUMP and 2 mM cCMP was added, respectively. Input designates mouse lung tissue before incubation. PKARIɑ was detected by immunoblotting.

Using cCMP or cUMP coupled to biotin, PKARIɑ was identified as a cCMP- and also cUMP- binding protein in A549 cells (Fig 5; S6 Fig) and mouse lung tissue (Fig 6; S7 Fig). Addition of cCMP and cUMP to the assays showed positive competition. No binding of PKARIɑ to biotin alone was detected neither using A549 cells nor mouse lung tissue. No binding of biotin to PKARIɑ was detected neither in A549 cells nor in mouse lung tissue. In A549 cells, stronger binding of cCMP to PKARIɑ was detected. In mouse lung tissue, comparable binding of PKARIɑ to cCMP and cUMP was observed.

## Discussion

Binding of cCMP to the regulatory subunits RIa and RIIa of PKA was validated using cCMP-agaroses as described by Hammerschmidt et al. [17]. Moreover, activation of PKARIɑ, PKARIIɑ and PKGIɑ by cCMP and cUMP was reported [15,16], but direct binding of cUMP to the regulatory subunits of PKA or binding of cCMP or cUMP to PKG was not shown. For the first time, we have detected binding of cUMP to the mentioned regulatory subunits of PKA and also binding of cCMP and cUMP to PKG. Further, we identified three different isoforms of PKG as cCMP- and cUMP-,expression of PKARIɑ in A549 cells and of PKARIIa in mouse lung tissue was apparently relatively low, as no expression of PKARIɑ and PKARIIɑ could be detected in the input samples.

With cCMP and cUMP coupled to biotin matrices we confirmed the binding of PKARIɑ to cCMP and cUMP. The specificity of interaction was shown by the effective displacement by an excess of cCMP and cUMP for agarose and biotin approaches, respectively. The high molecular weight signal in Fig 4, lane 6 could be a RI dimer. Bindung between cAMP and RI dimer was previously described by Brennan et al. (2006) after affinity purification with cAMP-agarose [21]. The pulldown of cytosolic proteins from bacteria that have relevance as signaling molecules had already been successfully demonstrated by Gundlach et al. with c-di-AMP-coupled to biotin [22]. To identify further proteins that may be important in cCMP and cUMP signaling pathways, further experiments with subsequent MS proteome analysis should be performed. Since biotin is smaller than agarose matrices, a divergent proteome should be identified due to differences between biotin and agarose matrices.

The agarose is attached to the ribose *via* the OH-group at the 2ˈ-position at the 2’-AHC-cUMP-agarose. By identification of PKARIɑ from mouse lung tissue using AA-cUMP-agarose, high coverages (71.6% and 66.9%) were obtained, whereas using 2’-AHC-cUMP-agarose only moderate coverages (5% and 13.9%) were measured. A comparable result was obtained for 2ˈ-AHC-cCMP-agarose and 4-AH-cCMP-agarose [17], which indicates the importance of the hydroxyl group at the ribose at the 2ˈ-position, shown by Kim et al. [13].

Clear differences in the performance of the two cUMP-agaroses can also be seen when constructing a cUMP-binding proteome from mouse lung tissue. For this purpose, all proteins were listed that were found by affinity purification with 5-AA-cUMP-agarose, which were neither present in the assays with EtOH-NH control agarose and not detectable in competition assays with cUMP (S8 Table). A comparative 2’-AHC-cUMP-agarose binding proteome in mouse lung lysate could not be generated using all criteria mention before. This result is also reflected in the generation of proteomes for the cell lines. While some potential cUMP-binding proteins could be filtered out with AA-cUMP-agarose, the results with 2’-AHC-cUMP-agarose were rare to nonexistent.

Direct binding between agarose or biotin to the nucleobase of a cNMP has the consequence that the structure of the nucleobase is changed (modified). The original structure of the nucleobase is maintained by binding between the matrices to the OH group of ribose. Nevertheless, a more efficient enrichment of binding partners was obtained with the nucleobase-modified molecules. This can be explained in consideration of the crystal structure of the regulatory subunit of PKA, published 1995 by Susan Taylor and co-workers [Su et al., 1995]. They showed that the beta-sandwich structure is highly conserved and identified the crucial amino acids of PKA that bind to cAMP. They found that the ribose and the cyclic phosphate residue fit into the binding pocket and the base tends to protrude upwards [23,24]. The models obtained with cAMP can be transferred analogously to cCMP and cUMP, because the molecules differ in the base, but not in the ribose and the cyclic phosphate residue. The structure, in particular the spatial accessibility and the size of the binding pocket, can also further influence the successful enrichment of proteins.

The biotin label was connected to cUMP at the 5-position via an 11-atom spacer and to the N^4^-position of cyclic CMP via a 14-atom spacer. These compounds should provide more effective results, analogous to the cCMP- and cUMP- agaroses. In order to verify this, assays would have to be carried out with the appropriate tools, which are not available until now.

These approaches could also be used to estimate binding strength by using competition experiments with the other cNMPs - cAMP and cGMP. In this way, one can also analyze whether identical binding sites are used. RIɑ has 2 binding domains A and B, these have different affinities for cAMP, first binding of cAMP to B leads to a conformational change which afterwards allows binding of cAMP to A [24]. It was not clear whether this mechanism was also relevant for cCMP and cUMP. However, it has already been shown that certain preferences of different cAMP analogues are also present at A or B sites. [25].

In addition to the proteins mentioned here, MS proteomics was used to identify other proteins that were not previously known to be binding partners of cCMP or cUMP (unpublished results). These results still need to be confirmed with independent experiments. Some proteins are low abundance proteins and detection with immunoblotting was not successful. With these proteins, experiments must be carried out with overexpressed proteins, whereby problems often arise with the negative controls due to the strong expression of the proteins of interest.

### Limitations of the study

It has long been known that cAMP is not evenly distributed in the cytosol within the established second messenger camp. By using specific probes that are present for both cAMP and cGMP, the compartmentalization could be analyzed in detail. Numerous studies using specific—mostly fluorescently labeled—probes have shown that cAMP is not evenly distributed in the cytosol. A nanodomain structure has been discovered by monitoring cAMP distribution in real time [26]. Appropriate tools are also available for investigating the cellular distribution of cGMP [27].

Since there are currently no corresponding probes for either cCMP or cUMP, no studies can be carried out to investigate the cellular distribution. Therefore, it is currently unknown whether a corresponding compartmentalization or a nanodomain structure exists and what physiological relevance cCMP and cUMP may have. This demonstrates the relevance of developing appropriate tools to study distribution. Unfortunately, the binding partners shown here are not suitable for this purpose, as they are known binding proteins of cAMP and cGMP. However, the matrices presented here could be very helpful in identifying specific binding proteins that were previously unknown. A further limitation of this study is that, although additional proteins were identified during these investigations, they could not be examined in more detail. Therefore, further, more detailed experiments are necessary.

### Future directions

Recently, the relevance of cCMP and cUMP in the immune system of bacteria for the defense of bacteriophages was demonstrated. Proteins were identified that bind specifically to cCMP or cUMP and not to cAMP or cGMP [12]. Specificity can be verified with these agarose and biotin matrices. The identified proteins (PycTM for cCMP and PycTIR for cUMP) could be suitable for the production of specific sensors, which would represent a leap forward in the research of cCMP and cUMP. Thus, from the chemical biology point of view, a promising start towards the development of biologically useful and selective probes for known and hitherto unknown cCMP-binding proteins has been made. The proteins mentioned under limitations that have not yet been identified as binding partners must be confirmed in further studies and their relevance in relation to cCMP and cUMP must be clarified.

Our results showed that cNMP-agaroses and biotin-cNMPs could be used in both – lysates of mammalian cells and tissue and also bacterial or fungi lysates – to identify binding partners. Therefore, based on the latest results from Tal et al. [12], it should be possible using these tools to find additional hitherto unknown binding proteins in lysates from bacteria and bacteria infected with bacteriophages.

## Supporting information

S1 Fig MS spectrum of PKG from mouse lung tissue with 5-AA-cUMP-agarose. (PDF)

S2 Fig MS spectrum of PKARIalpha from mouse lung tissue with 5-AA-cUMP-agarose. (PDF)

S3 Fig Original blot of Fig 2. ɑPKG western blot from mouse lung tissue after affinity chromatography with cCMP- and cUMP-agaroses.
(PDF)

S4 Fig Original blot of Fig 3. ɑPKARIɑ western blot from A549 cell lysate after affinity chromatography with cCMP- and cUMP-agaroses.
(PDF)

S5 Fig Original blot of Fig 4. ɑPKARIIɑ western blot from mouse lung tissue after affinity chromatography with cCMP- and cUMP-agaroses.
(PDF)

S5A Fig Original blot of Fig 4. ɑPKARIIɑ western blot from mouse lung tissue after affinity chromatography with cCMP- and cUMP-agaroses (longer exposition time).
(PDF)

S6 Fig Original blot of Fig 5. ɑPKARIɑ western blot from A549 cell lysate after affinity chromatography with cCMP and cUMP biotin matrices.
(PDF)

S7 Fig Original blot of Fig 6. ɑPKARIɑ western blot from mouse lung tissue after affinity chromatography with cCMP and cUMP biotin matrices.
(PDF)

S8 Table Proteome from mouse lung tissue using 5-AA-cUMP-agarose. Criteria: Proteins not detectable with EtOH-NH control agarose and not detectable in competition assays with cUMP. psm: peptide-spectrum match. For a peptide-spectrum pair, this value represents the probability that the peptide with a specific sequence was recorded in the experimental spectrum.
(PDF)

## Abbreviations

ACN: acetonitrile
cAMP: adenosine 3´,5´-cyclic monophosphate
cGMP: guanosine 3´,5´-cyclic monophosphate
cCMP: cytidine 3´,5´-cyclic monophosphate
cNMP: 3´,5´-cyclic nucleoside monophosphate
cUMP: uridine 3´,5´-cyclic monophosphate
HCN: hyperpolarization-activated cyclic nucleotide
LTQ: linear trap quadrupole
MS: mass spectrometry
PKA: cAMP-dependent protein kinase
PKARIɑ: regulatory subunit of PKA, type Iɑ
PKARIIɑ: regulatory subunit of PKA, type IIɑ
PKGIɑ: cGMP-dependent protein kinase Iɑ
PKGIβ: cGMP-dependent protein kinase Iβ
PKGII: cGMP-dependent protein kinase II
PDE: phosphodiesterase
TFA: trifluoroacetic acid
