# Supplementary figures and images for: Identification of cCMP- and cUMP-binding proteins using cCMP and cUMP coupled to agarose and biotin matrices
Source: PLoS One. 2025 Oct 14;20(10):e0333904. doi: 10.1371/journal.pone.0333904 (PMC12520408; doi:10.1371/journal.pone.0333904)

S1\_Fig

MS spectrum of PKG from mouse lung tissue with 5-AA-cUMP-agarose

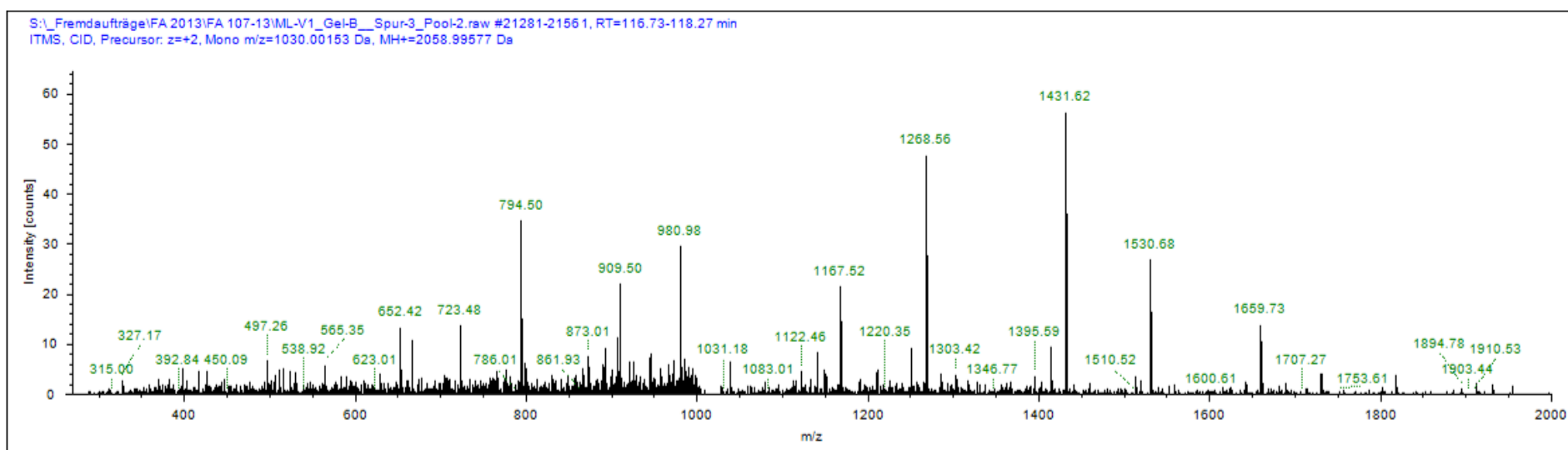

Supplement: S1 Fig — (PDF) [file pone.0333904.s001.pdf]

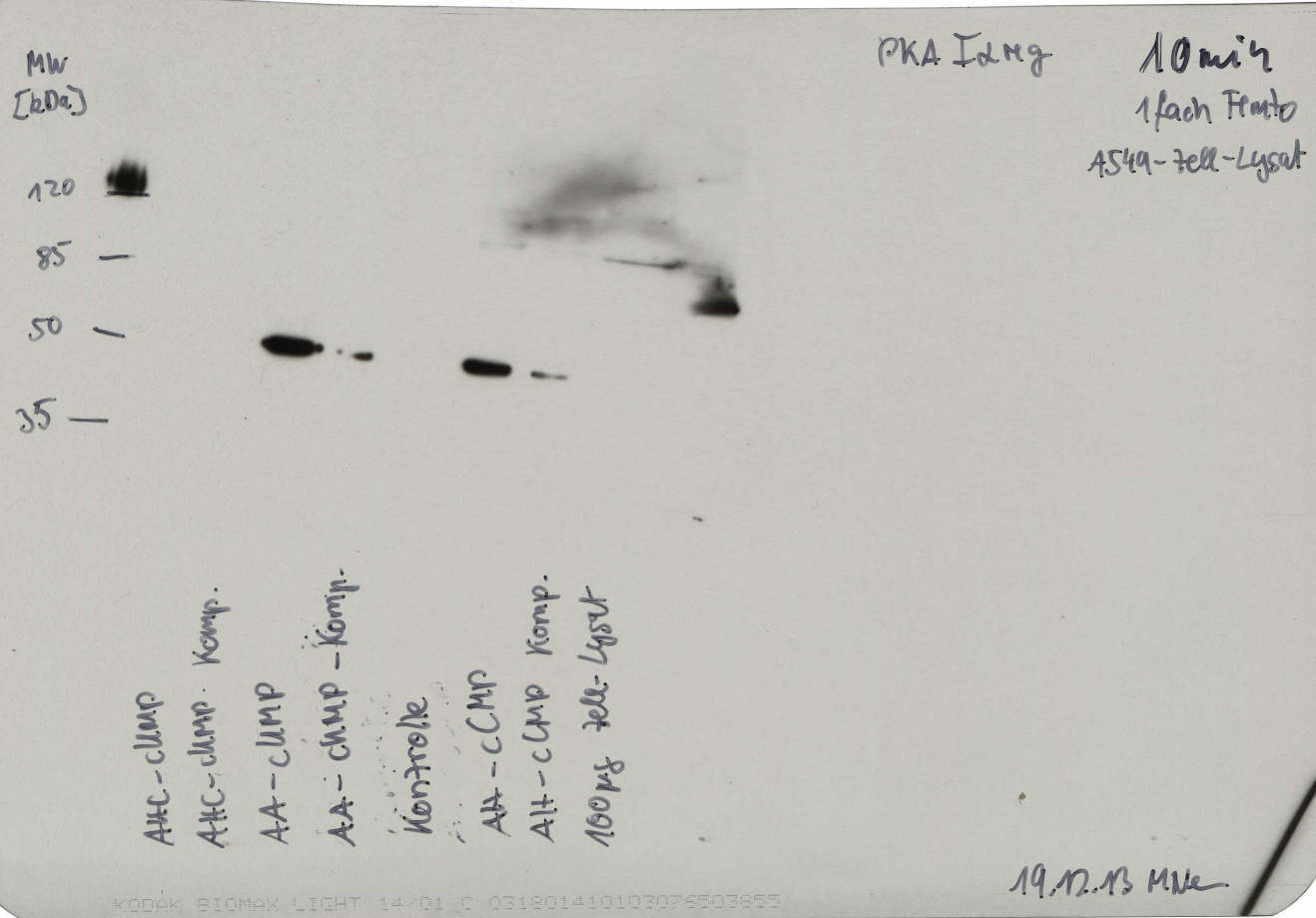

original blot: Fig.3

Supplement: S4 Fig — ɑPKARIɑ western blot from A549 cell lysate after affinity chromatography with cCMP- and cUMP-agaroses. (PDF) [file pone.0333904.s004.pdf]

kDa

70

55

35

1

2

3

4

5

6

7

original blot: Fig.5

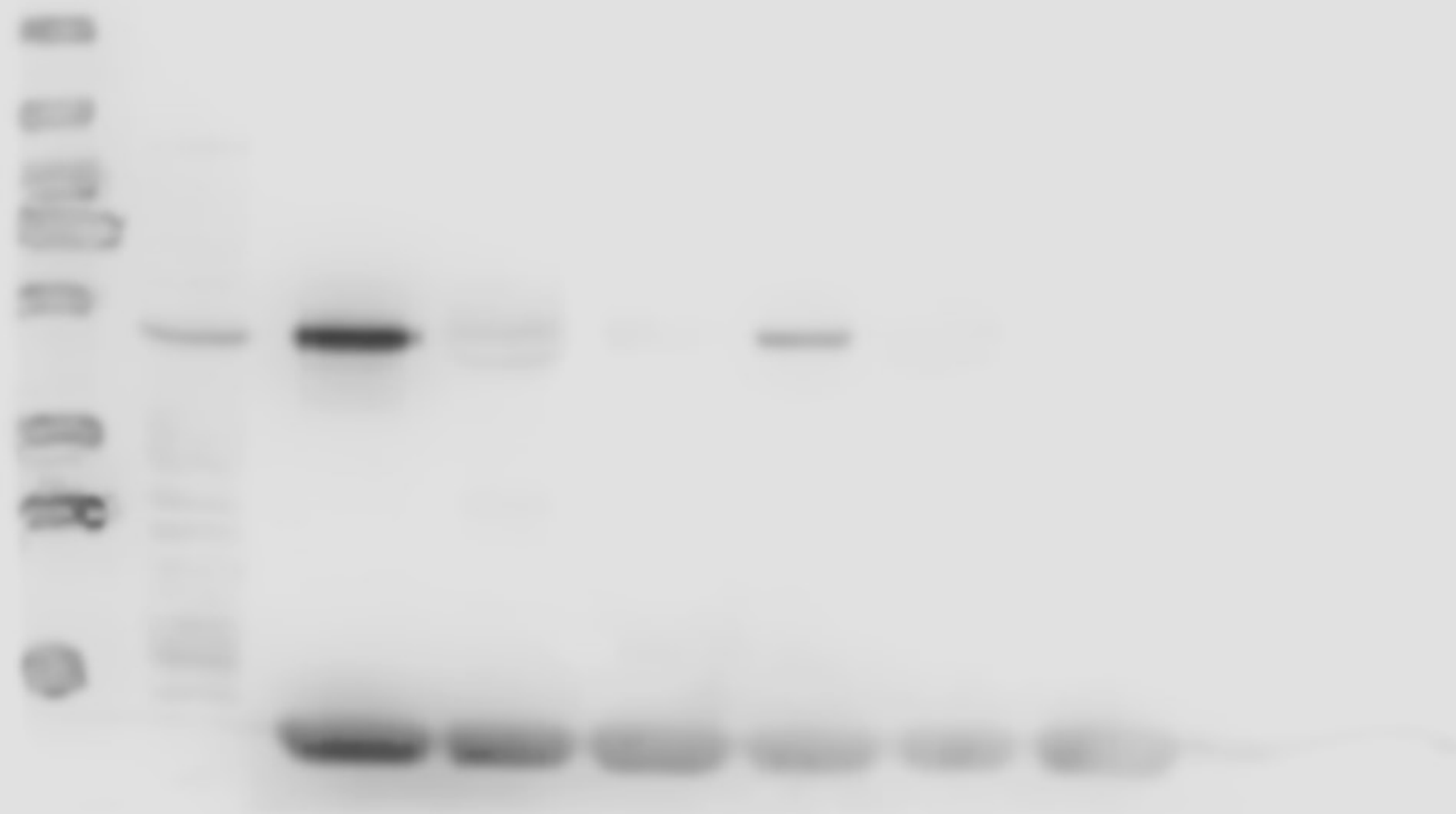

Supplement: S6 Fig — ɑPKARIɑ western blot from A549 cell lysate after affinity chromatography with cCMP and cUMP biotin matrices. (PDF) [file pone.0333904.s007.pdf]
