# Supplementary material for: Identification of cCMP- and cUMP-binding proteins using cCMP and cUMP coupled to agarose and biotin matrices
Source: PLoS One. 2025 Oct 14;20(10):e0333904. doi: 10.1371/journal.pone.0333904 (PMC12520408; doi:10.1371/journal.pone.0333904)

S2_Fig

MS spectrum of PKARIalpha from mouse lung tissue with 5-AA-cUMP-agarose

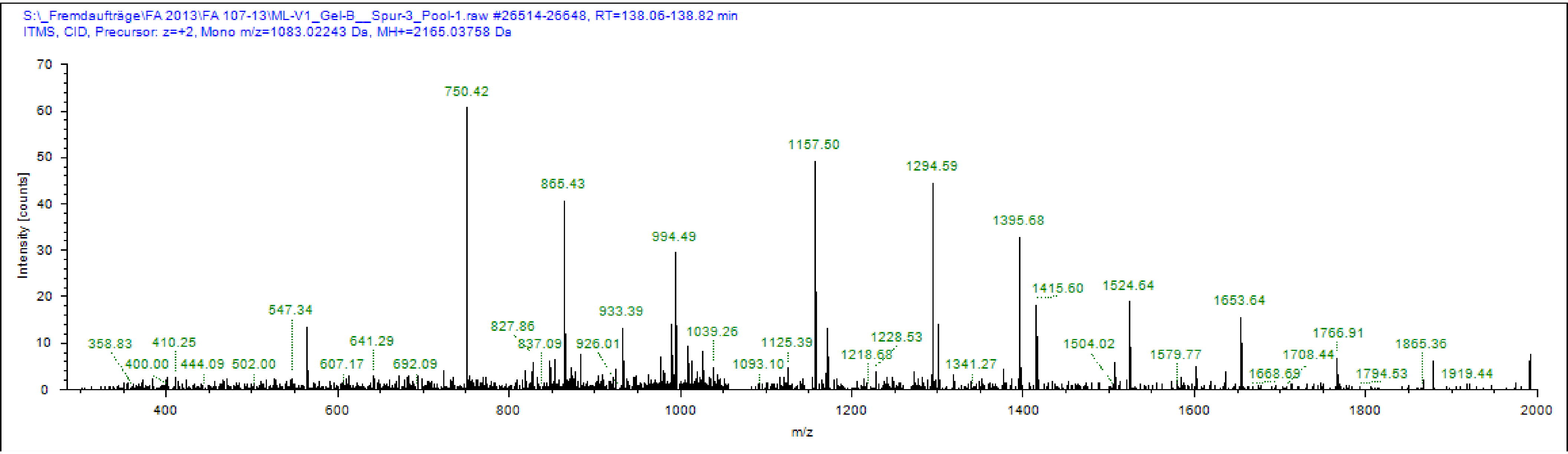

Supplement: S2 Fig — (PDF) [file pone.0333904.s002.pdf]
