# Supplementary material for: Identification of cCMP- and cUMP-binding proteins using cCMP and cUMP coupled to agarose and biotin matrices
Source: PLoS One. 2025 Oct 14;20(10):e0333904. doi: 10.1371/journal.pone.0333904 (PMC12520408; doi:10.1371/journal.pone.0333904)
Supplement: S5A Fig — ɑPKARIIɑ western blot from mouse lung tissue after affinity chromatography with cCMP- and cUMP-agaroses (longer exposition time). (PDF) [file pone.0333904.s006.pdf]

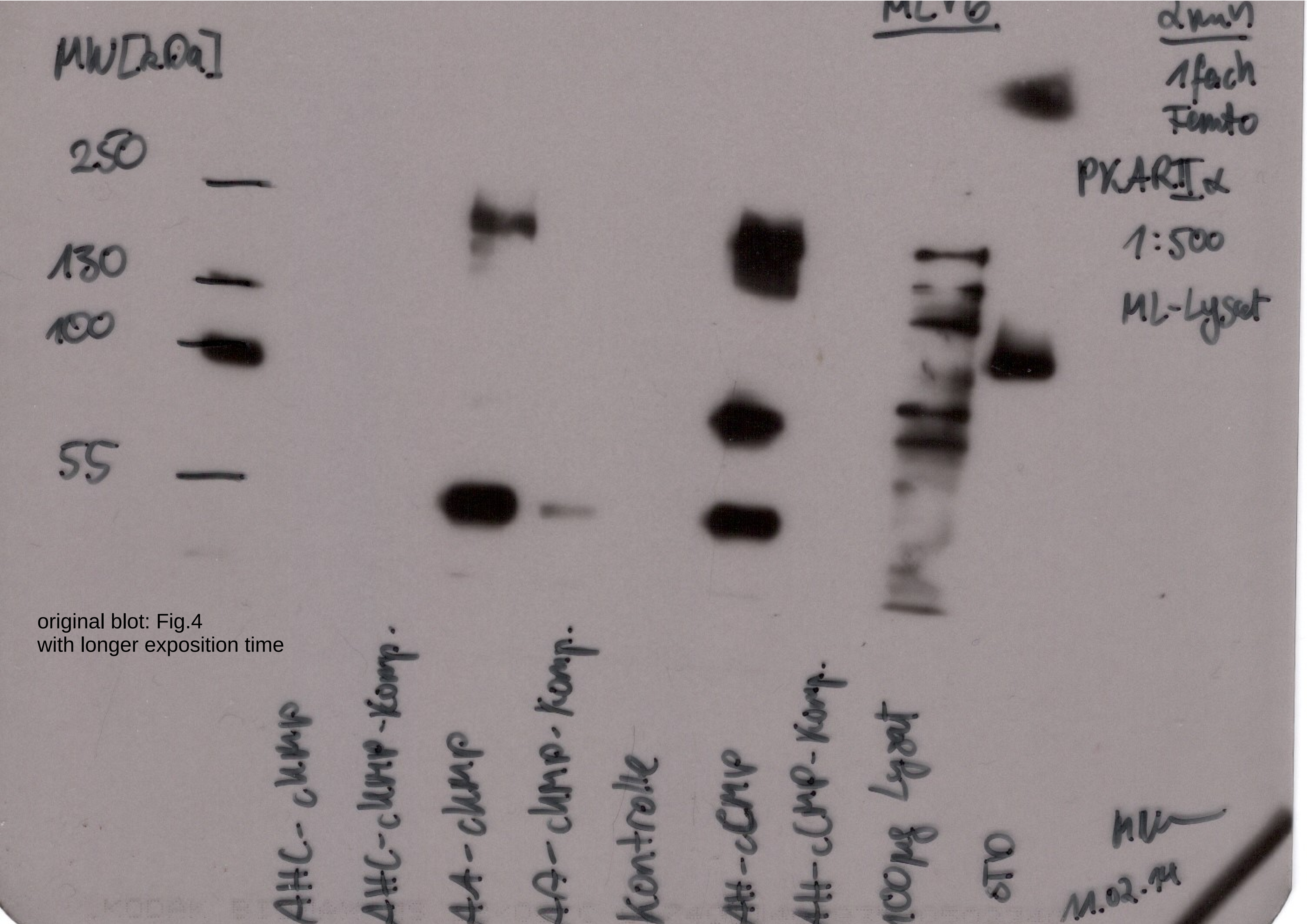

original blot: Fig.4
with longer exposition time
