# Supplementary material for: Identification of cCMP- and cUMP-binding proteins using cCMP and cUMP coupled to agarose and biotin matrices
Source: PLoS One. 2025 Oct 14;20(10):e0333904. doi: 10.1371/journal.pone.0333904 (PMC12520408; doi:10.1371/journal.pone.0333904)
Supplement: S7 Fig — ɑPKARIɑ western blot from mouse lung tissue after affinity chromatography with cCMP and cUMP biotin matrices. (PDF) [file pone.0333904.s008.pdf]

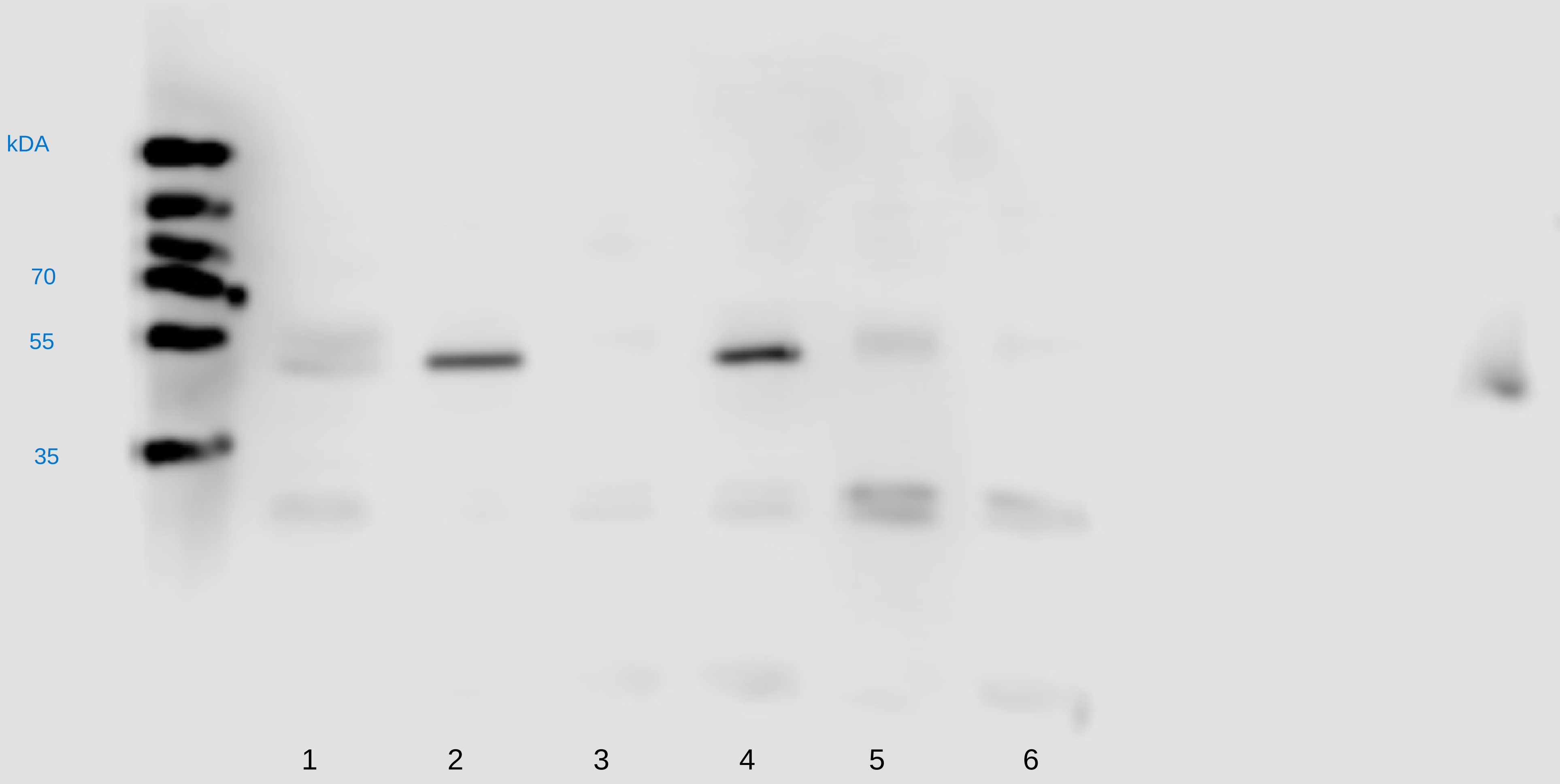


original blot: Fig. 6
