# Supplementary material for: Identification of cCMP- and cUMP-binding proteins using cCMP and cUMP coupled to agarose and biotin matrices
Source: PLoS One. 2025 Oct 14;20(10):e0333904. doi: 10.1371/journal.pone.0333904 (PMC12520408; doi:10.1371/journal.pone.0333904)
Supplement: S8 Table — Criteria: Proteins not detectable with EtOH-NH control agarose and not detectable in competition assays with cUMP. psm: peptide-spectrum match. For a peptide-spectrum pair, this value represents the probability that the peptide with a specific sequence was recorded in the experimental spectrum. (PDF) [file pone.0333904.s009.pdf]

S8_Table: Proteome from mouse lung tissue using 5-AA-cUMP-agarose

Accession	protein	Coverage (%)	# peptide	# psm
E9QPH0	cGMP-dependent protein kinase	26.33	20	55
E9Q070	Uncharacterized protein	13.25	2	4
D3Z7U0	Annexin	4.70	2	5
O35682	Myeloid-assciated differentiation marker	7.19	2	6
P10852	4F2 cell-surface antigen heavy chain	4.94	2	3
P26645	Myristolated alanine-rich C-kinase substrate	11.00	2	4
P355564	Calnexin	7.78	4	4
P47740	Fatty aldehyde dehydrogenase	4.96	2	2
P54116	Erythrocyte band 7 integral membrane protein	6.34	2	4
P59242-2	Isoform 2 od Cingulin	1.86	2	2
P61164	Alpha-centractin	7.98	3	5
P68254-2	Isoform 2 of 14-3-3 protein theta	28.40	8	25
P69181	cAMP-dependent protein kinase catalytic subunit beta	21.94	10	15
P633328-2	Isoform 2 of Serine/threonine-protein phosphatase 2B catalytic subunit alpha isoform	14.87	7	10
Q3TZZ7	Extended synaptotagmin-2	3.08	2	3
Q3U0V1	Far upstream element-binding protein 2	2.81	2	3
Q3U7R1-2	Isoform 2 of extended synaptotagmin-1	4.14	3	4
Q61696	Heat shock 70 kDa protein 1A	12.32	7	21
Q62470-3	Isoform 3 of Integrin alpha-3	4.60	4	4
Q7TMM9	Tubulin beta-2A chain	24.27	10	48
Q7TN79	A-kinase anchor protein 7 isoform gamma	19.11	7	22
Q8BFZ9	Erlin-2	7.06	2	6
Q8BH78	RTN4	25.56	5	6
Q8R3G9	Tetraspanin-8	14.89	4	12
Q8VDM4	26S proteasome non-ATPase regulatory subunit 2	5.84	4	5
Q9EQZ6-2	Isoform2 of Rap guanine nucleotide exchange factor 4	2.87	2	2
Q9WTQ5-2	Isoform 2 of A-kinase anchor protein 12	10.13	11	17
Q9WVC3	Caveolin-2	12.35	2	5

N=2

Criteria: Proteins not detectable with EtOH-NH control agarose and not detectable in competition assays with cUMP

psm: peptide-spectrum match

For a peptide-spectrum pair, this value represents the probability that the peptide with a specific sequence was recorded in the experimental spectrum.
